# Modification of Ordinary Concrete Using Fly Ash from Combustion of Municipal Sewage Sludge

**DOI:** 10.3390/ma13020487

**Published:** 2020-01-19

**Authors:** Gabriela Rutkowska, Piotr Wichowski, Małgorzata Franus, Michał Mendryk, Joanna Fronczyk

**Affiliations:** 1 Institute of Civil Engineering, Warsaw University of Life Sciences—SGGW, Nowoursynowska 166, 02-787 Warsaw, Poland; gabriela_rutkowska@sggw.pl; 2 Institute of Environmental Engineering, Warsaw University of Life Sciences—SGGW, Nowoursynowska 166, 02-787 Warsaw, Poland; piotr_wichowski@sggw.pl (P.W.); michal_mendryk@outlook.com (M.M.); 3 Faculty of Civil Engineering and Architecture, Lublin University of Technology, Nadbystrzycka 40, 20-618 Lublin, Poland; m.franus@pollub.pl

**Keywords:** concrete, sewage sludge combustion, fly ash, compressive strength of concrete, heavy metals leaching

## Abstract

This article focuses on the impact of fly ash from the combustion of municipal sewage sludge (FAMSS) as a cement additive in the amounts of 5%, 10%, 15%, 20% and 25% (by mass) on selected concrete properties. In the course of the experimental work, water penetration depth and compressive strength measurements were made at various periods of curing (from 2 to 365 days). In addition, the potential impact of FAMSS on the natural environment was examined by determining the leachability of heavy metals. FAMSS-modified concretes showed small values of water penetration depth (lower than 50 mm), as well as good compressive strength (reaching minimum class C30/37 after 130 days of maturing)—similar to the compressive strength obtained for conventional concrete. In addition, the partial replacement of cement with FAMSS has environmental benefits, expressed as a reduction in CO_2_ emissions. In addition, study has shown that compliance with environmental requirements is associated with heavy metal leaching.

## 1. Introduction

The cement production process can be harmful to the natural environment, emitting large amounts of greenhouse gases due to the high-temperature (1450 °C) process of burning cement clinker and the CO_2_ emissions resulting from the decarbonization of raw material [1]. Therefore, issues related to improving energy efficiency, protecting the environment and using waste as a raw material are the basic challenges of this industry [2,3].

According to the Yearbook of International Statistics data [4], the production of cement in the world amounted to over 1.67 billion tons in 2000, and 4.07 billion tons in 2013. As a result of this production, in 2000 the cement industry emitted 1.4 billion tons of greenhouse gases (converted to CO_2_) into the atmosphere, which accounted for 3% of global greenhouse gas emissions and about 5% of the CO_2_ emissions resulting from human activities; in 2013, however, cement production increased and amounted to 4.07 billion tons [3,4,5]. According to Statista (The Statistics Portal for Market Data) [6], global cement production in 2030 is expected to increase to 4.83 billion metric tons. China, India and the United States are among the three major cement producers in the world [6], while Poland is currently the third cement producer in Europe after Germany and Italy. The Central Statistical Office 2019 [7] data shows that, in 2018, cement production in Poland increased by 6% compared to 2017 and reached the level of 18,940 million tones. In 2001, CO_2_ emissions per 1 ton of clinker were 988 kg [8]. Thanks to the latest technological solutions, in 2018 the product emission was reduced to 807 kg CO_2_ per ton of clinker. However, it is impossible to produce clinker (and therefore also to produce cement) without emissions caused by the decomposition of about 525 kg CO_2_ per ton of clinker [9].

In cement technology, and mainly in the concrete technology in Europe, siliceous and calcareous fly ashes from the combustion of black coal and brown coal are widely used [10,11]. Their application in cement composites reduces carbon dioxide emissions and environmental pollution, as well as saves natural resources and fossil fuels [12,13,14,15,16,17,18,19,20]. It should be noted that the wide application of fly ashes from black coal and brown coal combustion in construction and building materials is mainly determined by the fineness, chemical and mineral composition, as well as pozzolanic activity—similar to cement. At the same time, fly ashes have a beneficial effect on the technical properties of concrete, improving its compressive and bending strengths, and frost resistance [21,22,23]. These factors make the production of ash concretes attractive to both producers and final consumers [2,24]. Nowadays, sewage sludge created in wastewater treatment plants is a waste with the code 190114 [25], the neutralization and management of which is a serious ecological issue. Sewage sludge combustion is one of the methods of its utilization, whose by-product is fly ash. Research conducted in recent years has been aimed at assessing the possibility of using fly ash from combustion of sewage sludge in concrete technology [12,26,27,28], and thus increasing waste recycling [26,29]. The physico-chemical properties and pozzolanic activity of fly ashes indicated the potential of this innovative additive [12,18,26,27,28,30]. However, the use of fly ashes as a partial cement substitute increases water demand and reduces the workability and density of concrete mixes [30,31]. Moreover, the use of fly ash from the combustion of sewage sludge in cement composites must be carefully controlled because of its physico-chemical properties, which can vary significantly depending on the type of wastewater entering the treatment plant, as well as on the technology used for sewage sludge processing [13,32,33]. The main mineral compounds present in sewage sludge fly ash are quartz (SiO_2_), calcium phosphate (Ca_3_(PO4)_2_), and hematite (Fe_2_O_3_), while the main elements are Si, Ca, Fe, Al, P and O [34]. Additionally, the considered fly ashes contain from 10 to 20% by mass of phosphates [34]. Phosphates can delay cement binding and thus affect the initial strength of concrete. Fly ash from the combustion of sewage sludge may also contain increased sulfate concentrations [35], which are derived from chemicals used in wastewater treatment processes [36].

Fontes et al [17] showed that cement mortars in which cement was replaced with FASS in an amount of 10% to 30% showed similar bending strength values to reference mortars. In addition, the partial replacement of cement with fly ash increased the porosity of concrete. Analyzing the results presented in the literature, a differentiated impact of fly ashes on compressive strength values can be noticed. In cement mortars—in which fly ash, together with cements containing a large amount of C3A, was used—no deterioration of compressive strength was observed after 28 days of maturing [37]. Donatello et al. [38] and Pan et al. [37] demonstrated that the replacement of 20% of Portland cement by fly ash from sewage sludge may result in a reduction in compressive strength of 24 to 52%. Cyr et al. [39] indicated similarly that the compressive and bending strengths after 28 days of curing decreased in comparison to ordinary mortar, while Chen et al. [16] observed a linear negative relationship between the compressive and bending strength of the analyzed mortars and the percentage of cement replacement by fly ashes. In the case of 10% cement replacement, the decrease in strength was smaller than for the sample with 25% of fly ash compared to the reference sample. However, in the studies of Monzo et al. [36,40], a moderate increase in the compressive strength of mortars with 15% sewage sludge ashes compared to comparative mortars was noted. The average increase in strength varied between 8.3–15.3%. According to Vouk et al. [41], the cement mortar mixtures with a content of fly ashes content up to 20% revealed similar and higher values of compressive and bending strength compared to reference mixtures depending on fly ash content. According to Baeza-Brotons [35], samples in which cement was exchanged for 5% of fly ash showed a slightly higher compressive strength, whereas samples with fly ash content above 5% showed a lower compressive strength than reference sample. However, it should be emphasized that the values of compressive strength were higher than 90% of the values determined for conventional concrete samples. Moreover, Donatello and Cheeseman [42] underlined that, in general, strength parameters may be improved by fly ash milling (grinding). On the other hand, an increase in sewage sludge ashes content causes a decrease in strength.

Taking the variability of physical and chemical composition of fly ash from the combustion of municipal sewage sludge (FAMSS) into consideration, their application can affect the features of concretes manufactured with their addition. The results of the investigations presented in the current paper are a continuation and extension of previous investigations [28]. The paper presents the test results of chemical and physical composition of fly ashes from municipal sewage sludge combustion extracted in the autumn of 2018, and the comparison with the results obtained for the ashes extracted in 2017. In an attempt to recognize the influence of FAMSS on the features of ordinary concretes, and to determine the compressive strength development in time—the concrete mature periods—the percentage share of fly ashes in the mix was extended.

## 2. Materials and Methods 

### 2.1. Aim and Scope of the Experiment

The main purpose of the research was to assess the properties of ordinary concretes with a modified composition. The modification consisted of the partial replacement of cement with fly ash from the combustion of municipal sewage sludge. For comparison, experiments were carried out for unmodified concretes and concretes, in which cement was replaced with FAMSS in the range of 5% to 25%. In addition to the technical properties of analyzed concretes, the leaching of selected substances (including heavy metals), as well as the environmental impact of the produced composites, were determined.

### 2.2. Preparation of Concrete Specimens 

Ordinary concrete samples were designed for testing in accordance with PN-EN 206 + A1: 2016-12 "Standard Concrete–Part 1: Requirements, properties, production and compliance" [43]. To perform the tests, concrete mixes of class C20/25 and thick-plastic consistency F2 were designed. The constant grain-size composition of fine aggregates, selected in sieve analysis, as well as of coarse aggregates, selected by consecutive iterations (Table 1), were maintained in all samples. The mix composition was designed using the three equations method of Bukowski [44]. To prepare the concrete samples, a natural aggregate was used with grain size from 0.125 to 16 mm, CEM I 32.5 Portland cement, as well as admixtures. As the mineral admixtures for the concrete mix, fly ashes from the fluidal combustion of municipal sewage sludge in the “Czajka” sewage treatment plant (Warsaw, Poland) were used. The FAMSS tested was produced in May 2018. In order to compare the properties of ordinary concretes produced in the traditional way and concretes containing FAMSS, two types of concrete samples were prepared:

1. Concrete without admixtures—CON,
2. Concrete mixed with fly ashes from combustion of municipal sewage sludge—FAMSS.

In the individual samples mixed with fly ashes, 5% (FAMSS 5%), 10% (FAMSS 10%), 15% (FAMSS 15%), 20% (FAMSS 20%) or 25% (FAMSS 25%) of a defined cement mass were replaced by the ashes. The maximum amount of fly ash included in the *k* value for CEM cement met the condition: fly ash FAMSS/cement C ≤ 0.33 (by mass). The concrete mix recipe per 1 m^3^ was established to comply with the assumption of ordinary concrete mix with use of the method of three equations. The proportions of concrete mix tested in the study are shown in Table 2. After forming, the specimens were cured by immersion in tap water at 18 ± 2 °C [43].

CEM I 32.5R Portland cement was used in the tests, in all exposure classes excluding classes XA2 and XA3, for which HSR sulphate cements are commonly used. Table 3 presents phase composition and chemical properties, while the physical properties of the FAMSS are presented in Table 4. 

### 2.3. Test Methods for Fly Ash 

In order to characterize the physical-chemical properties and activity of FAMSS, and to investigate the possibility of the modification of concretes made with their participation, their chemical composition, mineral composition, grain size and pozzolanicity were examined.

The chemical composition of fly ash was determined on an Epsilon 3 (Panalytical, Almelo, The Netherlands) spectrometer using the X-ray energy dispersion fluorescence (XRF) method. The test was performed in the measuring range of Na-Am elements on an apparatus equipped with an Rh X-ray tube (9 W, 50 kV, 1 mA), a 4096-channel spectrum analyzer (Panalytical), 6 measuring filters (Cu-500, Cu-300, Ti, Al-50, Al-200, Ag) and a high resolution solid state SDD detector (50 µm thick Be window) cooled with a Peltier’s cell. 

The mineral composition of the fly ashes was determined using powdered X-ray diffraction (XRD) tests with a Panalytical X’pertPRO MPD X-ray diffractometer (Panalytical) with a PW 3020 goniometer (Panalytical). A Cu copper lamp (CuKa = 1.54178 Å) was used as the source of X-ray emission. X’Pert Highscore software was used to process diffraction data. The identification of mineral phases was based on the PDF-2 Release 2010 database, formalized by JCPDS-ICDD.

The grain size analysis was based on laser diffraction using a Mastersizer 3000 analyzer (Malvern Instruments, Malvern, Worcestershire, UK). The measurement was carried out in a dispersing liquid (demineralized water) in the presence of an ultrasonic probe in order to break up larger aggregates of the tested samples. Grains with equivalent diameter sizes from 0.1 to 1000 µm were analyzed. The morphology and chemical composition in the micro area of the main components of the tested materials was determined using the SEM Quanta 250 FEG scanning electron microscope (FEI), equipped with a chemical composition analysis system based on X-ray energy dispersion—EDS (Energy Dispersive X - Ray Spectroscopy, EDAX) [28].

The pozzolanic activity of fly ash was carried out according to PN-EN 450-1: 2012 [46] and the ASTM C379-65TP [47] method as well as guidelines from the literature [48]. 

### 2.4. Test Methods for Concrete Mixes and Concrete

In order to characterize the properties of the concrete mixes tested, the following tests were carried out: consistency of concrete mixes by the fall cone method [49], bulk density by the mass and volume measurement method [50], and air content by the pressure method [51]. Compressive strength tests were performed according to the guidelines contained in PN-EN 12390-3: 2011 [52] at various curing periods (after 2, 7, 28, 56, 90, 130, and 365 days) on samples of dimensions 150 mm × 150 mm × 150 mm. The compressive strength tests were performed in the H011 Matest hydraulic test machine (Matest, Brembate Sopra, Italy). In the statistical analysis, standard deviation, coefficient of variation and total uncertainty for the recommended confidence interval p = 0.95 were determined for 6 tested samples. In accordance with the PN-EN ISO/IEC 17025 standard and the ISO procedure adapted for building materials, the unscaled sclerometric method was used to assess the expanded (total) uncertainty [15,53]. To test the significance of differences between the compressive strength after selected maturing periods of the reference sample and samples containing FAMSS, a one-way ANOVA variance analysis was performed. The obtained values of compressive strength were adopted as the dependent variable, while the quality factor was the FAMSS content in the individual samples. As part of testing the technical parameters of concrete, the depth of water penetration under pressure was also determined in accordance with EN 12390-8:2019-08 [54]. The test was carried out for 6 samples for each type of concrete after 28 days of maturing. Statistical analyses of the results were performed using Microsoft Excel and Statistica 13.3 software.

Moreover, concrete containing 5, 10, 15, 20 and 25% of fly ashes as well as the control sample were tested for heavy metals leaching using European standards [55]. Samples of crushed concrete were sieved through 6.3 mm and 0.63 mm meshes, and further tests were carried out for fractions smaller than 0.63 mm and for the fraction of particles between 0.63 and 6.3 mm. Distilled water (pH = 6.7, electrical conductivity EC = 0.0270 mS/cm) was added to each sample at a 10:1 liquid/solid phase ratio and mixed in a rotary shaker (GFL, Burgwedel, Germany) at 20 rpm for 24 h at room temperature (21 ± 2 °C). After standing for 30 min, the leachate was filtered through a 0.45 mm filter paper and analyzed for (Cd, Cr, Cu, Ni, Pb, Zn, As, Se, and Ba) concentrations using the method of inductively coupled plasma atomic emission spectrometry (ICP-AES Thermo Scientific iCAAP 6500 spectrometer, Waltham, MA, USA). Additionally, the concentrations of chlorides and sulphates were determined by titration using chemical reagents and digital burettes (Jencons Digitrate, Bedfordshire, UK) with the accuracy of ±0.01 mL, the concentration of orthophosphate using the colorimetric method with molybdovanadate (DR-6000 spectrophotometer, Hach, Loveland, CO, USA), as well as pH and EC (Eijelkamp 18.50.01 multimeter, Giesbeek, Nederland), turbidity (by 455 nm, 2100N IS turbidimeter, Hach, USA) and color (DR-6000 spectrophotometer, Hach, USA) of the eluate were determined. Triplicate samples were analyzed (n = 3) and their average value was calculated.

## 3. Results and Discussion

### 3.1. Properties of Fly Ash and Concrete Mix

**Figure 1:**
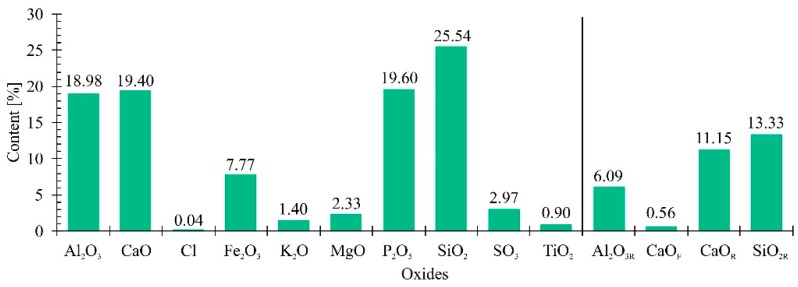
Chemical composition of fly ash from the combustion of municipal sewage sludge (FAMSS) (subscript “F” refers to free oxides and subscript “R” refers to reactive oxides).

The results of the composition analysis, with additional values of free calcium oxide (CaO_F_) content, and reactive aluminum, calcium and silica oxides (Al_2_O_3R_, CaO_R_ and SiO_2R_, respectively) of FAMSS are presented in Figure 1. Silica, phosphorus, aluminum and calcium oxides constituted the largest percentage in the fly ash oxide composition. It was observed that the sum of silica dioxide (25.54% of SiO_2_), aluminum oxide (18.98% of Al_2_O_3_) and iron oxide (7.77% of Fe_2_O_3_) contents in FAMSS was lower than in conventional ashes, and did not meet the requirements of the PN-EN 450-1 + A1: 2012 standard (minimum 65% total) [46], which is consistent with the results obtained for FAMSS produced in May 2017 [29]. However, the sum of SiO_2_, Fe_2_O_3_ and Al_2_O_3_ contents in FAMSS from 2018 (52.29%) was higher than in FAMSS from 2017 (35.40%) tested by Rutkowska et al. [28]. Differences in the chemical composition of the applied ashes can affect the investigated strength properties of the concretes. The chemical components which possibly can affect the improvement of strength properties of the concretes are mainly free SiO_2_ and CaO. In the ash applied in 2018, their percentage shear was higher than in that used in 2017. However, the strength results presented in this work do not correlate with the results of the chemical composition. In the previous work, the growth in the percentage share from 10 to 15% did not cause any significant changes in the mechanical strength of the obtained concrete. Nevertheless, the result was ca. 42 ÷ 46 MPa. In this work, the growth in the percentage share caused growth in the mechanical strength. However, the comparable result with the ash from 2017 (~45 MPa) was obtained only for the 15% addition. This is namely the visible difference between the ashes used in both works. This can be evoked by the fact that there is much more Al_2_O_3_ in the ash from 2018 than in that from 2017, which significantly changes the SiO_2_/Al_2_O_3_ ratio. In the previous work, this ratio amounted to 1.6 more than the value obtained in 2018 (1.35). The higher SiO_2_/Al_2_O_3_ ratio means more free loadings, which possibly positively affect the concrete hydration process. It is the result of replacement of trivalent elements (Al^3+^) by tetravalent ones (Si^4+^).

Moreover, previously mentioned standards [42] require the content of the reactive SiO_2_ to be higher than 25%, reactive CaO lower than 10%, MgO lower than 4%, and Na_2_O lower than 5% by weight. For the tested FAMSS, the content was 13.33, 11.15, 2.33 and 0%, respectively. The set requirements were not met for reactive SiO_2_ and reactive CaO; the standard applies, however, to siliceous fly ashes obtained during the combustion of coal or co-combustion of coal and waste. In addition, compared to calcareous and siliceous fly ash, it has been noted that FAMSS contains significantly higher amounts of phosphate, which also exceed the required value of a maximum 100 mg/kg [46]. This is due to phosphorus removal from the wastewater and its accumulation in sewage sludge. It is worth emphasizing that these results are consistent with those presented by Tarko et al. [56]. Additionally, the loss of ignition, expressing the content of unburned coal in a given sample of FAMSS sludge, was 0.53%. It is connected with the technology of combustion of municipal sewage in the fluidized bed furnace, and with the combustion temperature exceeding 850 °C.

**Figure 2:**
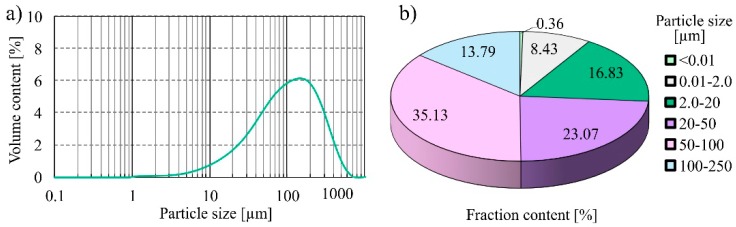
Particle size distribution curve (**a**) and volume distribution of individual particle fractions (**b**) in FAMSS.

**Figure 3:**
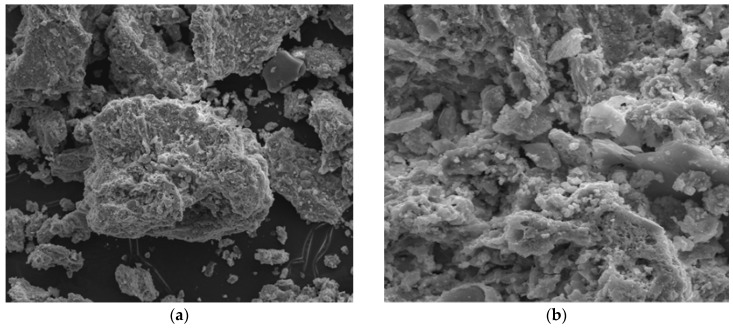
SEM images of FAMSS: (**a**) magnification 1000×; (**b**) magnification 4000×.

The particle size distribution of FAMSS tested was monomodal (Figure 2). It can be observed that the maximum particle size was 230 µm. Particles with a diameter of 2 to 250 µm account for 88.82% by volume. The particle fractions from 50–100 µm and 20–50 µm belonged to fractions with the largest percentage (by volume), and constituted 35.13% and 23.07%, respectively. For comparison, the particle size distribution of fly ash from another period of operation of the sewage sludge incinerator (May 2017) achieved a maximum value of 100 μm, and grains with a diameter of 2 to 250 μm constituted 91% of the volume [28]. The tests confirm that the strength parameters of concretes produced on the basis of FAMSS are influenced by the grain size of the additive. The most favorable parameters were obtained with thicker ash particle size used by Rutkowska et al. [28,57]. The specific surface area of the FAMSS was 2560 cm^3^/g, while the specific density was 1.826 kg/dm_3_. It should be emphasized that, according to Monzo et al. [40], the size of the additive is an important parameter for determining the strength of cement and ash mortars. Larger ash particle sizes cause a decrease in the compressive and tensile strengths of the material. Quartz and anhydrite dominate, with the addition of phosphates in the form of apatite and fluoroapatite, in the mineral composition of fly ashes from the combustion of municipal sewage sludge [58]. Additionally, the SEM images of FAMSS were presented in Figure 3.

The pozzolanic activity of fly ash according to ASTM C379-65T [48] is determined on the basis of the SiO_2_ and Al_2_O_3_ content. According to the literature, the total content of SiO_2_ and Al_2_O_3_ above 20% indicates the pozzolanic character of the tested samples [59]. Based on the investigations, it was found that the total content of reactive silica oxide (13.33%) and reactive aluminum oxide (6.09%) in the FAMSS tested was 19.42% (Figure 1). The pozzolanic activity desirable of FAMSS after 28 days of curing was 68.5% (required ≥75%), while after 90 days it was 79.3% (required ≥85%); therefore, FAMSS does not meet the requirements of PN-EN 450-1: 2012 [46]. However, the value of the analyzed parameter exceeded the required values (85%) after 180 days of curing. It is believed that the phosphorus contained in the fly ashes delays the process of binder hydration [60]. The results obtained in our research confirm those presented by Ferreira et al. [61], Monzo et al. [30] and Merino et al. [31], that the pozzolanic activity of FAMSS reaches the standard value (85%) after a longer period of curing. Higher values of pozzolanic activity of siliceous fly ash may be determined by the content of alkali in its composition. Potassium oxide plays a major role in this case. According to the literature [48], potassium and aluminum oxide, and thus the K_2_O/Al_2_O_3_ ratio, determine the activity of fly ashes. The content of K_2_O and Al_2_O_3_ in siliceous fly ash tested by Rutkowska et al. [61] was 0.2% and 27.8%, and in FAMSS it was 1.40% and 18.98% (Figure 1), respectively. However, according to Yusuf and Noor [62], the pozzolanic properties and chemical composition (silica, iron, calcium, aluminum) of fly ashes from the combustion of sewage sludge are analogous to the mineral additives that are commonly used. Hubbard and Dhir [48] introduced the pozzolanic potential desirable (K_2_O/Al_2_O_3_·10), which, depending on the proportion of oxides, subdivides fly ashes into three classes. An index of 0.73 was obtained for the FAMSS tested, which classifies the material into medium reactive class (the second class of reactivity). 

### 3.2. Physical Properties of Concrete Mix and Hardened Concrete

Based on consistency tests of the concrete mix using the fall cone method for the reference concrete and samples containing 10% and 15% of fly ash, a dense plastic consistency was obtained, while for the remaining samples a wet consistency was attained (Table 5). The lowest air content in the reference sample was 3.1%, while the highest for the FAMSS 25% sample was 4.6% (Table 5). An increase in the air content was also observed, along with an increase in the FAMSS content. As specified in PN-EN 206 + A1: 2016-12 [43], concretes with air content above 4% can be used for exposure class XF (environmental impact—freezing/thawing). The density of the concrete mix attained values from 2319 (FAMSS 25%) to 2387 kg/m^3^ (CON) and was in the range characteristic for ordinary concrete (2000–2600 kg/m^3^).

According to Monzo et al. [30] and Merino et al. [31], partial replacement of cement with FAMSS reduces the workability of the concrete mix; however, it does not threaten environmental safety. This is due to the fact that the fly ash grains have an irregular structure and high consistency. In addition, based on the analysis of the course of fly ash hydration, it was found that a binder meeting the requirements for cements could be obtained when fly ash replaces 20% of the mineral components used in the production [60,63]. In practical applications, it is recommended to use superplasticizers [31,60].

### 3.3. Mechanical Properties of Concrete

The average compressive strength at different curing periods with a marked standard deviation and the expanded uncertainty for concrete samples with a variable content of FAMSS is shown in Table 6. The reference concrete samples after two days of curing achieved a compressive strength of 21.92 MPa, while the highest value of this parameter equal to 23.22 MPa was observed for FAMSS 20% (increase by 5.9%) and the smallest value equal to 19.01 MPa for FAMSS 5% (decrease by 13.3%). After the next measuring period (7 days of curing), the CON sample achieved a strength of 37.82 MPa. The FAMSS 5% concrete achieved a 29.9% decrease in strength compared to the reference concrete. The smallest compressive strength after 28 days of curing—equal to 35.61 MPa—was obtained for concrete samples, in which 5% of cement was replaced with fly ash (FAMSS 5%), while the highest strength, equal to 42.58 MPa, was obtained for concrete samples, in which 20% of cement was replaced with fly ash (FAMSS 20%). Compared to the reference concrete, the decrease in the compressive strength of the FAMSS 5% concrete was 13.9%, while the increase in the FAMSS 20% concrete was 2.9%. Similar relationships were observed in subsequent curing periods: the lowest compressive strength was obtained for FAMSS 5% and the highest for FAMSS 20%. The decrease in compressive strength compared to reference concrete was in the range of 11% to 15%, while the increase was in the range of 1 to 7%. Moreover, it was noticed that all concretes made after 28 days of maturation had the C20/25 concrete class assumed during the design. After 90 days of curing, concrete samples with the lowest ash content (FAMSS 5%) obtained class C25/30, while the remaining concrete samples were class C30/37. After 365 days of curing, the CON and FAMSS 10%, 15% and 20% concretes were classified as class C35/45, while concretes with 5 and 25% ash content were in class C30/37. It should be emphasized that the extension of the maturing period reduced the differences in compressive strength values. Based on the obtained test results, it was found that by replacing cement with FAMSS, it is possible to obtain concretes with compressive strength very similar to the reference concrete. Nevertheless, the partial replacement of cement with FAMSS has environmental benefits, which include a reduction in CO_2_ emissions (Table 7). Assuming CO_2_ emissions of 807 kg per 1 ton of clinker, when replacing 25% of cement with FAMSS, about 200 kg less CO_2_ will be transferred into the atmosphere. Rutkowska et al. [28] proved that the most favorable conditions were obtained with the addition of 10% of FAMSS (from May 2017) to the concrete mixture. It must be noted that, although the fly ash came from the same installation, it differed in chemical composition and physical properties (e.g., particle size distribution). Moreover, the compressive strength after 28 days of concrete maturing with 10% of thicker ash from 2017 obtained 43.6 MPa—and, after 56 days, obtained 46.2 MPa—whereas with 10% of ash from 2018 the compressive strengths were 37.8 MPa and 44.62 MPa, respectively. Research presented in the literature [30,34,60,62,64] concerned the impact of fly ash on the strength parameters of concrete and mortar. It was found that replacement of cement with FAMSS up to 15% had a positive effect on the compressive strength of the concretes produced [56]. However, Monzo et al. [30] proved that mortars containing up to 15% of ashes show compressive strength comparable to conventional mortars. Moreover, in the case of using additional treatments, such as sample maturation at elevated temperatures or wet ash milling before adding to the mortar, the ash content may be increased to 30% of the binder mass [30]. Baeza-Brotons et al. [35], however, observed that concrete samples (containing up to 20% of FAMSS) achieved similar mechanical properties to conventional samples without ash after 28 days of maturation, and that the addition of ash significantly reduced water absorption. Chang et al. [65] in turn showed that the addition of FAMSS in an amount over 10% reduced the compressive strength of the resulting material. Chen et al. [16] confirmed that both concrete and mortar produced by replacing parts of sand and parts of cement with fly ash in amounts of over 10%, the cement showed less bending and compressive strength compared to materials produced without addition. On the other hand, mortar containing up to 10% of fly ash had a compressive strength similar to conventional mortar [16]. The coefficient of variation, together with standard deviation, as a measure of dispersion, is used to test the degree of variation in the value of a variable. The highest standard deviation (2.67 MPa) was obtained for a concrete sample, in which cement was exchanged with FAMSS in an amount of 5% (FAMSS 5%) after 28 days of curing, while the smallest deviation equal (0.42 MPa) was obtained by reference concrete samples (CON). The coefficient of variation ranged from 1.0% (CON) to 12% (FAMSS 5%). This indicates a low variability of characteristics and homogeneity of the studied population. Furthermore, the quality of the concrete tested is very good. The total uncertainty after 2 days of curing in the tested series at the recommended confidence level p = 0.95 was in the range of 1.44 MPa (FAMSS 15%) to 3.66 MPa (FAMSS 5%) while, after 7 days, it was in the range of 1.9 MPa (FAMSS 10%) to 2.95 MPa (FAMSS 25%). After 28 days, the uncertainty was 1.52 MPa for CON to 4.14 MPa for FAMSS 5%; after 56 days of curing in the range of 1.86 MPa for FAMSS 10% to 4.55 MPa for FAMSS 15%; after 90 days of curing in the range of 2.26 MPa for FAMSS 25% to 3.73 MPa for FAMSS 5%; while after a year of curing, in the range of 2.31 MPa for CON to 3.89 MPa for FAMSS 5%.

**Figure 4:**
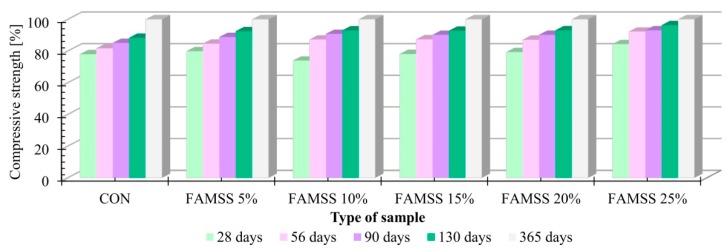
Relative changes in compressive strength.

After the first measurement, i.e., after 2 days of curing, the tested concrete obtained from 39% (FAMSS 10%) to 44% (FAMSS 25%)—and after 7 days from 58% (FAMSS 15%) to 71% (CON)—of the final strength determined after 365 days of maturation. After the next period of cement maturation (28 days), the reference concrete had 78% of the final (annual) compressive strength, while in concretes made with FAMSS, the compressive strength accounted for 74–84% (Figure 4). After 56 days of curing, the reference concrete was characterized by the largest increase in compressive strength. In the interval between 28 and 56 days of curing, the concretes with the addition of fly ash, despite a small increase in strength, had already obtained from 84 to 92% of the final strength. The obtained test results may indicate the pozzolanic activity of FAMSS. Figure 4 presents the relative changes of compressive strength in relation to maturation period for individual concrete, only for maturing times equal to and longer than 28 days, i.e., for times enabling achieving operational parameters. According to Autors [59,63], the presence of ashes at an amount of 25% by weight slows the grout setting process and weakens the compressive strength of mortars and concretes in comparison to composites made using only Portland cement. However, faster compressive strength growth was observed in our research for FAMSS 25% compared to control sampling.

The results of the analysis of variance (ANOVA) carried out for data obtained for individual maturation periods are presented in Table 8. The obtained values of the significance level p lower than 0.05 indicated the statistical significance of the differences and, therefore, the significant impact of the FAMSS content on the compressive strength of the concrete mixtures.

Based on the conducted tests of the depth of water penetration under pressure, a decreasing depth was observed along with the increase in the percentage of FAMSS (Table 9). According to the EN 12390-8:2019-08 [54] standard, concrete is defined as waterproof when the maximum depth of water penetration under pressure measured at the fractures of tested samples is less than 50 mm. The samples of reference concrete (CON) obtained the largest depth of water penetration, equal to 25.2 mm. The concrete samples with the addition of FAMSS reached values from 12.6 mm (FAMSS 25%) to 20.7 mm (FAMSS 5%). The water penetration depth did not exceed 50 mm in any sample tested, which should be considered a satisfactory result. The coefficient of variation ranged from 7% (CON) to 11% (FAMSS 15% and FAMSS 20%). The smallest standard deviation (1.04 mm) was obtained for a concrete sample, in which cement was exchanged with FAMSS in an amount of 25% (FAMSS 25%), while the highest deviation equal (1.97 mm) was obtained by the sample FAMSS 10%. This indicates the low variability of characteristics and homogeneity of the studied population. 

### 3.4. Leachability 

The chemical and physical characteristic of eluates from the crushed concrete samples is presented in Table 10. The pH of the eluates indicates a tendency for the value of this parameter (in the range of 11.91 ± 0.04 to 12.32 ± 0.05) to decrease with rising proportion of FAMSS in the cement mix. It may be influenced by the pH of FAMSS itself, which was 9.75 ± 0.08 [28]. However, no difference in the pH between the particle fractions was observed for all the tested concrete mixes. That relationship was observed for electrical conductivity; higher EC values of the eluate were observed for the particle fractions smaller than 0.63 mm. A significant decrease in the value of this parameter was also observed, along with the increasing content of FAMSS. Based on the test results obtained, it is not possible to clearly determine the impact of the percentage of FAMSS and the fraction of crushed concrete on the color and turbidity of the eluate. 

Studies have confirmed the assumption that the addition of FAMSS increases the leaching of phosphorus compounds (orthophosphates) relative to reference concrete (Table 10). It was also observed that for concrete samples containing from 10 to 20% of FAMSS, leaching of PO_4_ was higher for particle fractions smaller than 0.63 mm. It could be due to the larger contact surface of distilled water with the tested material. The concentration of sulfates in the eluate was only higher than the detection limit of the method for concrete samples with a grain size of 0.63–6.3 mm, containing 20% and 25% of FAMSS. The test results indicated an inverse relationship between the chloride leaching rate and the FAMSS content in concrete, but only in the range of 5% to 20% of FAMSS. Further increase in fly ash content caused a slight increase in chloride leaching. However, the addition of FAMSS increased the chloride concentration in the eluate in relation to the reference concrete. It should be emphasized that even the highest concentration of Cl in the eluate observed (34 mg/L) was lower than the values obtained by [28] for concretes containing conventional siliceous and calcareous ashes.

For all analyzed samples, the concentration of As, Cd, Ni, Pb and Se in the eluates was lower than the detection limit (Table 11). The barium concentration increased with the FAMSS content in concrete and with the grinding of concrete. The largest differences between fine (0.63 mm) and coarse fractions (0.63–6.3 mm) were observed for FAMSS 20% and FAMSS 25%. Chromium was reported in samples containing 5% to 15% FAMSS; for the control sample and for concretes containing 20% and 25% of ash, the Cr concentration was lower than the detection limit. The grain size of the crushed concrete had no significant effect on the degree of leaching of this metal. Copper was washed out from all samples, including the control sample. The Cu concentration decreased with the ash content; however, for FAMSS 5% and FAMSS 10% samples with particles of <0.63 mm, the Cu concentration was higher than for reference concrete. Compared to the results of the research presented by Rutkowska et al. [28], the concentrations obtained for FAMSS 15% were lower for SO_4_, Cl and Ba and higher for Cu and Cr. Moreover, leachability tests confirmed the lack of stability of fly ash properties produced in the same municipal sewage sludge processing installation. According to Jiao et al. [65], the leaching of heavy metals from FAMSS is controlled by several processes, e.g., the leaching of Cr and Cd is influenced by their dissolution, and the leaching of Pb, Zn and Cu by precipitation and sorption processes. 

In summary, it should be noted that the ashes studied are environmentally safe, because the sum of heavy metals washed out from concrete containing up to 25% FAMSS is less than 1.512 mg/L (the maximum permissible value is 10 mg/L). The concentration of Mo, Hg and Sb was not included in this value; however, according to previous studies for FAMSS [28], their concentration was below the limit of detection. Moreover, metal concentrations in the eluate did not exceed the limit values for inert waste. The achieved research results are consistent with the results obtained by other research centers. Previous research taken up on the topic [13,16,18,20] has concentrated on the using possibilities of fly ashes for construction purposes, taking into account both environmental and technical criteria. It should also be emphasized that at any stage of their application, there must be no risk to people employed in working with waste and users, and—additionally—these activities should be economically profitable.

## 4. Conclusions

Fly ashes are a very important additive modifying ordinary concrete and new generation concrete. However, when considering the use of fly ashes in concrete production, the physico-chemical composition, fineness and pozzolanic activity of the additives should be taken into account. Based on the presented research, the assessment of concrete modification with fly ash from the combustion of municipal sewage sludge was carried out and the following conclusions can be drawn:

1. The addition of the analyzed fly ashes in amounts up to 25% by mass does not cause the deterioration of compressive strength of concrete in relation to reference concrete. The best strength parameters compared to the reference concrete were obtained for samples containing 10% to 20% FAMSS.
2. The maturation time of the produced composites had a significant impact on the obtained results. The slow increase in the strength of concrete containing FAMSS could be caused by the presence of phosphorus delaying the cement hydration process.
3. All concretes obtained the same class of C30/37 and higher C35/45 (FAMSS 20%) after 130 days of maturing, while the reference concrete (CON) and concretes with FAMSS in the amount of 10% (FAMSS 10%), 15% (FAMSS 15%) and 20% (FAMSS 20%) after 365 days of maturing achieved an even higher class of C35/45.
4. The depth of water penetration under pressure for all samples was less than 30 mm, which met standard requirements.
5. The pozzolanic activity of FAMSS did not meet the applicable requirements after 28 days (≥75%) and 90 days (≥85%) of curing. FAMSS reached the required values of pozzolanic activity (85%) after a longer period of curing, which allowed it to be classified as active mineral additives.
6. Calcium, silica, phosphorus and aluminum oxide are the largest percentage of oxides in FAMSS samples. The sum of silica oxide, aluminum and iron oxides content does not meet the requirements contained in existing standards. However, there are no regulations regarding the physical and chemical properties of fly ashes from combustion of municipal sewage sludge.
7. Based on the sum of heavy metals leached and the concentration of individual heavy metals concentration in the eluate it may be concluded that concrete containing FAMSS in the range of 5% to 25% is environmentally safe. However, studies of the physical and chemical properties of FAMSS have shown their variability in time. Therefore, further research on the impact of this variability on the technical parameters of concretes containing FAMSS seems necessary.

## Figures and Tables

**Table 1:** Percentage content of the aggregates selected by the iterations.

Fraction	Fraction Mixing Percentage Ratio (for Sand and Gravel)			Grain Composition	
	I stage	II stage	III stage	Sand	Gravel
0.0–0.125	-		39	1.44	0.56
0.0125–0.25				16.23	6.33
0.025–0.50				30.46	11.88
0.50–1.0				28.54	11.13
1.0–2.0				23.33	9.10
2.0–4.0	-	34	61	-	20.74
4.0–8.0	46	66		-	18.52
8.0–16.0	54			-	21.74

**Table 2:** Concrete mix proportions by weight.

Specification	Mass of Concrete Ingredients (kg/m^3^)			
	Water	Aggregate	Cement	Fly Ash
CON	167.23	1885.18	376.27	0
FAMSS 5%	167.23	1885.18	357.46	18.81
FAMSS 10%	167.23	1885.18	338.64	37.63
FAMSS 15%	167.23	1885.18	319.83	56.44
FAMSS 20%	167.23	1885.18	301.02	75.25
FAMSS 25%	167.23	1885.18	282.20	94.07

**Table 3:** Chemical and phase composition of CEM I cement [45].

Component (unit)	Content	Content of CEM I Mineral Phases (mass%)
Loss in ignition (%)	3.18	C_3_S—61.8
Sulphate content SO_3_ (%)	3.20	C_2_S—12.3
Chloride content Cl (%)	0.05	C_3_A—7.5
Alkali content as Na_2_Oeq (%)	0.71	C_4_AF—4.0
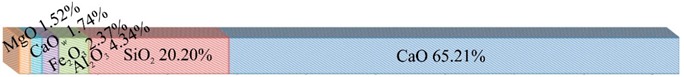		

**Table 4:** Physical parameters of cement [45].

Physical Parameters of Cement	Unit	Average Values
Specific surface area according to Blaine	(cm^2^/g)	3330
Start of setting time	(min)	218
Compressive strength after 2 days	(MPa)	21.0
Compressive strength after 28 days	(MPa)	49.8

**Table 5:** Physical properties of the concrete mix.

Type of Concrete	Consistency (mm)	Air content (%)	Density (kg/m^3^)
CON	90 (S2)	3.1	2387
FAMSS 5%	39 (S1)	2.8	2362
FAMSS 10%	67(S2)	3.3	2353
FAMSS 15%	58 (S2)	3.8	2338
FAMSS 20%	34 (S1)	4.1	2327
FAMSS 25%	27 (S1)	4.6	2319

**Table 6:** Compressive strength of concrete with FAMSS.

Type of Concrete	Period of Curing (days)	f_cm_^1^ (MPa)	f_c min_^2^ (MPa)	f_c max_^3^ (MPa)	S ^4^ (MPa)	V ^5^ (-)	Up ^6^ 0.95 (MPa)	Concrete Class Obtained
CON	2	21.92	20.59	23.44	1.37	0.06	2.16	C12/15
	7	37.82	36.42	39.12	1.51	0.04	2.57	C25/30
	28	41.36	40.62	42.16	0.42	0.01	1.52	C25/30
	56	43.06	41.68	43.94	0.93	0.02	1.99	C30/37
	90	44.88	43.40	45.97	1.27	0.03	2.40	C30/37
	130	46.60	45.75	47.82	0.83	0.02	1.99	C30/37
	365	52.72	51.52	53.94	1.01	0.02	2.31	C35/45
FAMSS 5%	2	19.01	17.36	20.46	1.97	0.10	2.99	C12/15
	7	26.52	25.01	27.98	1.49	0.06	2.39	C16/20
	28	35.61	33.47	37.45	2.67	0.08	4.14	C25/30
	56	37.74	36.12	40.37	1.45	0.04	3.73	C25/30
	90	39.62	38.10	41.73	2.35	0.06	3.73	C25/30
	130	41.24	40.12	42.67	1.49	0.04	2.61	C30/37
	365	44.58	42.77	46.12	2.43	0.05	3.89	C30/37
FAMSS 10%	2	20.21	18.07	21.74	2.43	0.12	3.66	C12/15
	7	34.52	33.10	35.49	1.02	0.02	1.90	C25/30
	28	37.84	36.54	39.12	1.28	0.03	2.29	C25/30
	56	44.62	43.62	45.63	0.75	0.02	1.86	C30/37
	90	46.46	45.10	47.94	1.36	0.03	2.55	C30/37
	130	47.56	46.36	48.74	1.04	0.02	2.21	C30/37
	365	51.12	49.86	52.33	1.12	0.02	2.38	C35/45
FAMSS 15%	2	21.66	20.86	22.98	0.84	0.04	1.44	C12/15
	7	30.66	29.79	31.24	1.30	0.04	2.19	C20/25
	28	41.11	40.06	42.18	0.78	0.02	1.72	C30/37
	56	45.96	43.93	47.85	2.89	0.06	4.55	C30/37
	90	47.48	46.10	49.54	2.10	0.04	3.50	C30/37
	130	48.85	46.46	50.62	3.10	0.06	4.88	C30/37
	365	52.61	50.98	53.42	1.24	0.02	2.54	C35/45
FAMSS 20%	2	23.22	21.80	24.50	1.62	0.07	2.52	C12/15
	7	32.16	31.05	33.40	1.38	0.04	2.32	C20/25
	28	42.58	41.47	44.70	2.15	0.05	3.49	C30/37
	56	46.73	45.42	48.36	1.48	0.03	2.69	C30/37
	90	48.43	47.00	49.90	1.54	0.03	2.79	C30/37
	130	49.94	48.82	51.74	1.59	0.03	2.88	C35/45
	365	53.64	52.33	54.85	1.55	0.03	2.91	C35/45
FAMSS 25%	2	20.65	19.10	21.79	1.22	0.06	1.93	C12/15
	7	29.40	28.15	31.22	1.88	0.06	2.95	C20/25
	28	39.16	37.85	41.45	2.53	0.06	3.98	C25/30
	56	42.83	41.23	44.00	1.42	0.03	2.54	C30/37
	90	43.21	42.00	44.65	1.17	0.03	2.26	C30/37
	130	44.70	43.46	45.98	1.09	0.02	2.10	C30/37
	365	46.40	45.25	47.95	1.53	0.03	2.74	C30/37

^1^ average compressive strength; ^2^ minimum compressive strength; ^3^ maximum compressive strength; ^4^ standard deviation; ^5^ coefficient of variation; ^6^ total expanded uncertainty for the confidence level (*p* = 0.95).

**Table 7:** Impact of FAMSS application on the reduction in CO_2_ emissions.

Type of Concrete	Mass of Concrete Ingredients (kg/m^3^)		CO_2_ Emission (kg/m^3^)	Reduction in CO_2_ Emission (kg/m^3^)
	Cement	Fly Ash		
CON	376.27	0	303.65	
FAMSS 5%	357.46	18.81	288.47	15.18
FAMSS 10%	338.64	37.63	273.28	30.37
FAMSS 15%	319.83	56.44	258.10	45.55
FAMSS 20%	301.02	75.25	242.92	60.73
FAMSS 25%	282.20	94.07	227.74	75.91

**Table 8:** Results of ANOVA analysis of compressive strength at various periods of concrete maturation.

Compressive Strength After	SS Effect	df Effect	MS Effect	SS Error	df Error	MS Error	F	p
2 days	65.69	5	13.14	39.13	30	1.30	10.07	0.000
7 days	471.92	5	94.38	34.84	30	1.16	81.27	0.000
28 days	200.81	5	40.16	39.13	30	1.30	30.79	0.000
56 days	309.35	5	61.87	43.40	30	1.45	42.77	0.000
90 days	313.25	5	62.65	38.88	30	1.30	48.34	0.000
130 days	296.37	5	59.27	38.94	30	1.30	45.66	0.000
365 days	417.74	5	83.55	39.52	30	1.32	63.43	0.000

**Table 9:** Depth of water penetration under pressure.

Parameter	Type of Concrete					
	CON	FAMSS 5%	FAMSS 10%	FAMSS 15%	FAMSS 20%	FAMSS 25%
Depth of water penetration (mm)	25.2	20.7	19.6	15.8	13.8	12.6
Standard deviation (mm)	1.7	1.92	1.97	1.80	1.57	1.04
Coefficient of variation (-)	0.07	0.09	0.10	0.11	0.11	0.08

**Table 10:** Chemical and physical parameters of eluates.

Type of Concrete	Fraction (mm)	pH	EC (mS/cm)	Color (mgPt)	Turbidity (NTU)	PO_4_ (mg/L)	SO_4_ (mg/L)	Cl (mg/L)
CON	<0.63	12.30 ± 0.08	8.43 ± 0.23	24 ± 15.5	4.02 ± 0.42	0.10 ± 0.00	nd	17.7 ± 1.4
	0.63–6.3	12.26 ± 0.11	7.55 ± 0.46	22 ± 11.3	3.55 ± 0.17	0.05 ± 0.07	nd	14.9 ± 1.2
FAMSS 5%	<0.63	12.28 ± 0.03	9.17 ± 0.11	23.5 ± 4.5	2.87 ± 0.96	0.10 ± 0.14	nd	34.0 ± 2.0
	0.63–6.3	12.27 ± 0.04	7.13 ± 0.76	20.5 ± 0.7	2.05 ± 0.10	0.10 ± 0.14	nd	32.6 ± 1.7
FAMSS 10%	<0.63	12.32 ± 0.05	8.05 ± 0.39	32 ± 2.8	3.95 ± 2.34	0.30 ± 0.07	nd	32.6 ± 1.5
	0.63–6.3	12.24 ± 0.03	7.63 ± 0.55	24.5 ± 2.1	6.68 ± 5.69	0.20 ± 0.00	nd	29.8 ± 1.7
FAMSS 15%	<0.63	12.22 ± 0.04	8.82 ± 0.16	25.5 ± 13.4	3.99 ± 3.13	0.30 ± 0.07	nd	26.9 ± 1.3
	0.63–6.3	12.13 ± 0.04	7.56 ± 0.12	25.5 ± 16.2	3.98 ± 2.77	0.15 ± 0.07	nd	25.8 ± 1.5
FAMSS 20%	<0.63	12.10 ± 0.03	3.16 ± 0.12	22 ± 5.7	3.05 ± 1.07	0.25 ± 0.07	nd	19.9 ± 1.3
	0.63–6.3	12.01 ± 0.05	3.54 ± 0.02	24.5 ± 3.5	3.86 ± 1.78	0.20 ± 0.07	1 ± 0.97	15.6 ± 1.4
FAMSS 25%	<0.63	11.99 ± 0.04	3.69 ± 0.62	27 ± 8.5	6.35 ± 5.42	0.35 ± 0.21	nd	21.3 ± 1.1
	0.63–6.3	11.91 ± 0.04	3.15 ± 0.84	40.5 ± 9.2	9.38 ± 1.26	0.5 ± 0.14	10 ± 1.2	18.4 ± 1.2

nd—not detected.

**Table 11:** Heavy metals concentration in eluates.

Sample	Heavy Metals (m/L)									
	As	Ba	Cd	Cr	Cu	Ni	Pb	Se	Zn	ΣHM
CON										
<0.63	<0.010	0,447	<0.002	<0.010	0.043	<0.005	<0.003	<0.010	<0.030	<0.086
0.63–6.3	<0.010	0,371	<0.002	<0.010	0.040	<0.005	<0.003	<0.010	<0.030	<0.083
FAMSS 5%										
<0.63	<0.010	0,601	<0.002	0.038	0.048	<0.005	<0.003	<0.010	<0.030	<0.119
0.63–6.3	<0.010	0,363	<0.002	0.034	0.033	<0.005	<0.003	<0.010	<0.030	<0.100
FAMSS 10%										
<0.63	<0.010	0,659	<0.002	0.038	0.046	<0.005	<0.003	<0.010	<0.030	<0.117
0.63–6.3	<0.010	0,421	<0.002	0.036	0.032	<0.005	<0.003	<0.010	<0.030	<0.101
FAMSS 15%										
<0.63	<0.010	0,660	<0.002	0.039	0.035	<0.005	<0.003	<0.010	<0.030	<0.107
0.63–6.3	<0.010	0,507	<0.002	0.033	0.036	<0.005	<0.003	<0.010	<0.030	<0.102
FAMSS 20%										
<0.63	<0.010	1.37	<0.002	<0.010	0.027	<0.005	<0.003	<0.010	<0.030	<1.440
0.63–6.3	<0.010	0.421	<0.002	<0.010	0.019	<0.005	<0.003	<0.010	0.044	<0.524
FAMSS 25%										
<0.63	<0.010	1.44	<0.002	<0.010	0.029	<0.005	<0.003	<0.010	<0.030	<1.512
0.63–6.3	<0.010	0.146	<0.002	<0.010	0.011	<0.005	<0.003	<0.010	<0.030	<0.200
